# Epidemiology and patterns of care for invasive breast carcinoma at a community hospital in Southern India

**DOI:** 10.1186/1477-7819-5-56

**Published:** 2007-05-23

**Authors:** Sambasivaiah Kuraparthy, Kumaraswamy M Reddy, Lakshmi Amancharla Yadagiri, Mutheeswaraiah Yutla, Phanindra Bobbidi Venkata, Sarma VS Kadainti, Ramasubba PV Reddy

**Affiliations:** 1 Department of Medical oncology, Sri Venkateswara institute of Medical Sciences, Tirupati, India; 2 Department of Pathology, Sri Venkateswara institute of Medical Sciences, Tirupati, India; 3 Department of General Surgery, Sri Venkateswara institute of Medical Sciences, Tirupati, India; 4 Department of Radiology, Sri Venkateswara institute of Medical Sciences, Tirupati, India; 5 Department of General Surgery, S.V. R.R. General hospital, Tirupati, India; 6 Department of Statistics, S.V. University, Tirupati, India

## Abstract

**Background:**

Breast cancer incidence in India is on rise. We report epidemiological, clinical and survival patterns of breast cancer patients from community perspective.

**Methods:**

All breast cancer patients treated at this hospital from July 2000 to July 2005 were included. All had cytological or histological confirmation of breast cancer. TNM guidelines for staging and Immunohistochemistry to assess the receptor status were used. Either lumpectomy with axillary lymph node dissection or Modified radical mastectomy (MRM) was done for operable breast cancer, followed by 6 cycles of adjuvant chemotherapy with FAC or CMF regimens to patients with pT >1 cm or lymph node positive or estrogen receptor negative and radiotherapy to patients after breast conservation surgery, pT size > 5 cm, 4 or more positive nodes and stage IIIB disease. Patients with positive Estrogen receptor or Progesterone receptor were advised Tamoxifene 20 mg per day for 3 years. Descriptive analysis was performed. Independent T test and Chi-square test were used. Overall survival time was computed by Kaplan – Meier method.

**Results:**

Of 1488 cancer patients, 122 (8.2%) had breast cancer. Of 122 patients, 96.7% had invasive breast carcinoma and 3.3% had sarcoma. 94% came from the rural and semi urban areas. Premenopausal women were 27%. The median age was 50 years. Stage I-6.8%, II-45.8%, III-22%, IV-6.8%, Bilateral breast cancer – 2.5%. The mean pT size was 3.9 cm. ER and PR were positive in 31.6% and 28.1% respectively. MRM was done in 93.8%, while 6.3% patients underwent breast conservation surgery. The mean of the lymph nodes dissected were 3. CMF and FAC regimens were used in 48.8% and 51.2% of patients respectively. FAC group were younger than the CMF group (43.6 yr vs. 54 yrs, P = 0.000). Toxicities were more in FAC than CMF group, alopecia (100% vs. 26.2%), grade2 or more emesis (31.8% vs. 9.2%), grade2 or more fatigue (40.9% vs.19%), anemia (43.1% vs. 16.6%). Median Survival for the cohort was 50.8 months. ER positive patients had better median survival (P = 0.05).

**Conclusion:**

MRM was the most frequent surgical option. CMF and FAC showed equivalent survival. FAC chemotherapy was more toxic than CMF. ER positive tumors have superior survival. Overall 3 year survival was 70 percent

## Background

Breast cancer is the most common cancer in the women of developed countries. Survival in breast cancer patients has improved substantially over the years as a result of multimodal treatment, comprising of local treatment by surgery and radiotherapy; systemic treatment by chemotherapy and hormonal therapy. Present research is focused on further refinement of each of these treatments in an attempt to preserve the organ and reduce the immediate and delayed toxicities. Studies on early stage breast cancer (EBC) from the developed countries have reported 20 year survival rates [1].

In India, without a significant reduction in carcinoma cervix, women are exposed to the increased risk of breast cancer, as demonstrated by the project "Development of An Atlas of Cancer In India", this study showed that age-adjusted incident rates of breast cancer was more than that of cervical cancer in 11 population based cancer registries (PBCR), with sole exception of a rural PBCR at Barshi [2,3].

Health care inequalities exist in India. As a result of inappropriate concentration of comprehensive cancer centers to metros, only a fraction of total breast cancer patients could access these services. It is therefore, not unusual to find suboptimal treatment of breast cancer in the rural and semi urban regions of India. Various studies on breast cancer published from India reflect the disease profile and treatment characteristics unique to the urban rich and the middle class patients. The breast cancer profile at community level is largely unrepresented. Breast cancer is the second most frequent at this hospital, situated and catering to the rural patients [4]. We report the epidemiology, clinical characteristics and treatment patterns from a rural perspective. 3 year survival is included.

## Methods

All breast caner patients who were treated at this hospital from July 2000 to July 2005 were included. All had cytological and/or histological confirmation of breast cancer. Patients were staged as per TNM guidelines. Chest-X ray, Ultrasound of liver was done for all, while Bone scan was done for Stage IIIB patients. Hormone receptor status was assessed by Immunohistochemistry, a quick score of 5 and above was considered positive. Cardiac status was assessed by Electrocardiogram and Echocardiogram for patients in anthracyline group.

### Surgery

Surgery, either lumpectomy with axillary dissection or Modified radical mastectomy was done upfront for operable breast cancer. Some patients, who had surgery at peripheral hospitals and private clinics, were also included after slide review and provided that they had completed adjuvant treatment and follow up at our hospital. Premenopausal women who do not attain menopause after chemotherapy were advised bilateral oopherectomy.

### Chemotherapy

All patients with pT >1 cm or axillary lymph node positive or estrogen receptor negative were given adjuvant chemotherapy. Adjuvant chemotherapy protocols included FAC (5 FU 600 mg/m2, Doxorubicin 60 mg/m2 and cyclophosphamide 600 mg/m2 given as i.v. infusion once every 21 days) or CMF regimens (Cyclophosphamide 600 mg/m2, Methotrexate 40 mg/m2 and 5 FU 600 mg/m2 given on day1 and day8 as i.v infusion repeated every 28 days). Maximum of 6 cycles of chemotherapy were given. CMF regimen was given to elderly patients, with clinical cachexia and economically underprivileged. Patients having locally advanced cancer were given neoadjuvant chemotherapy, which was followed by surgery. Patients with positive Estrogen receptor and/or Progesterone receptor were advised Tamoxifene 20 mg per day for 3 years, after completion of chemotherapy.

### Radiotherapy

Radiotherapy was given to all those who underwent breast conservation surgery. Additionally, patients having pT >5 cm, four or more positive axillary lymph nodes, close or positive surgical margins and all stage IIIB patients were given radiotherapy. Patients without data on pT size and axillary nodes were advised adjuvant radiotherapy. Radiotherapy was given after completion of chemotherapy. Palliative radiotherapy for painful skeletal lesions was also given in metastatic setting.

### Statistics

Descriptive analysis was performed for baseline demographics, response rates and toxicities. Independent T test was used to compare two chemotherapy groups. Chi-square test was used to explore the association between the categorical variables. Survival time was calculated from the date of diagnosis to the last follow up known to be alive or death. Kaplan – Meier method and log – rank test was used to compute overall survival.

## Results

Of 1488 cancer patients treated during this period, 122 (8.2%) had breast cancer. Of 122 breast cancer patients 118 (96.7%) patients had invasive breast carcinoma, while 4 (3.3%) patients had sarcoma. Sarcoma patients were not included for analysis. Clinical features are provided in Table 1. Only 14.4% of women had meaningful education of degree and above. 55% of women came from the rural background, while 39% of them were from semi urban areas. Premenopausal women comprised 27% of the cohort. Family history of any solid tumor was reported in 17% of patients (table 2). Mean age at first delivery was 21 years. Mean reproductive period (Age at Menopause minus Age at Menarche) was 32 years. Mean weight of the whole cohort was 56.8 kg, while the mean Body Mass Index (BMI) was 24.1.

**Table 1:** Clinical features (Figures in parenthesis show percentage)

Age (Median)	50 years
range	(23–72)
Stage	
I	8 (6.8)
II	54 (45.8)
III	26 (22.0)
Metastatic	8 (6.8)
Bilateral Breast Ca	3 (2.5)
Axillary Lymph node	
Positive	42 (35.6)
Negative	29 (24.6)
Data missing	47 (39.8)
Estrogen Receptor	
Positive	22(18.6)
Negative	35 (29.7)
Data missing	61 (51.7)
Progestorone Receptor	
Positive	19 (16.1)
Negative	37 (31.4)
Data missing	62 (52.5)
Surgery	
Breast conservation	6 (5.1)
Mastectomy	90 (76.3)
Chemotherapy	
FAC	44 (51.2)
CMF	42 (48.8)
Radiotherapy	50 (42.4)

**Table 2:** Frequencies of risk factors for breast cancer

Parameter	Yes (%)	No (%)	Missing Data (%)
menopause	70 (59)	32(27)	16(14)
Abortion	29(25)	71(60)	18(15)
Oral contraceptive pill use	4(3)	112(95)	2(2)
Breast fed	78(66)	2(2)	38(32)
Family history of any cancer	20(17)	85(72)	13(11)
Education(Degree and above)	17(14)	69(59)	32(27)
Diabetes	25(21)	60(51)	33(28)
mixed diet	91(77)	12(10)	15(13)

Early breast cancer and as well as metastatic breast cancer was marginal at 6.8% each. Of 96 operated patients, only 14 (14.6%) had data on pT size. The mean pT size was 3.9 cm. Fifty-seven (48.3%) had information on receptor status. ER was positive in 38.6% of patients and PR was positive in 33.3% patients. Only 15.8% of patients were both ER and PR positive.

Of 96 patients who had surgery, 55 (57.3%) patients had surgery at tertiary care hospital, while 42.7% patients had surgery at peripheral hospitals and private clinics. Mastectomy was done in 93.8% patients, while 6.3% patients underwent breast conservation surgery (BCS). Seventy-one (73.9%) had information on axillary lymph nodal status, out of these 33 (46.5%) patients had information on the number of nodes dissected. The mean of the lymph nodes dissected were 3. Of 96 patients who underwent surgery, 86 (89.6%) patients received chemotherapy. Of 86 patients who received chemotherapy, 11(12.8%) were in neoadjuvant setting. CMF and FAC regimens were used in 48.8% and 51.2% of patients respectively. The characteristics of the two chemotherapy groups are listed in the table 3. The mean of chemo cycles received was 4.9 and 5.3 in CMF and FAC groups respectively (P = 0.27). The distribution of estrogen receptor positive patients were not significant between two chemotherapy groups (chi-square P = 0.5). Thirty-eight patients received hormonal therapy. Mean duration of Tamoxifene treatment was 13.5 months.

**Table 3:** comparison of chemotherapy groups

variable	group	N	Mean	Stan Dev	t	mean Difference	Pvalue
Age	CMF	42	54.0	9.42	4.99	10.4	0.000
	FAC	44	43.6	9.97			
Weight (kg)	CMF	40	54.0	11.11	-2.42	-5.4	0.018
	FAC	42	59.4	8.91			
Body mass index	CMF	40	23.1	4.36	-1.75	-1.6	0.083
	FAC	42	24.6	3.75			
Lymph nodes	CMF	14	3.0	1.25	0.28	0.2	0.784
	FAC	14	2.8	1.81			
Tumor size (clinical)	CMF	32	6.1	2.61	0.67	0.7	0.505
	FAC	40	5.4	1.98			
Hemoglobin	CMF	30	11.6	1.93	0.03	0.0	0.977
	FAC	35	11.6	1.68			
Albumin	CMF	18	4.0	0.31	1.09	0.1	0.283
	FAC	22	3.8	0.50			

### Toxicity

Toxicities (Table 4) were assessed in patients who received chemotherapy. FAC regime was particularly more toxic across all the events reported. Mucositis was seen in 4.8% and 13.6% of CMF and FAC group patients respectively (P = 0.15). Alopecia was seen in all FAC patients and in 26.2% of CMF group (P = 0.000). Grade2 and more, emesis was seen in 9.2% of CMF and 31.8% of FAC patients (P = 0.05). Grade3 and above, nausea was seen in 2.4% of CMF and 9.1% of FAC patients (P = 0.12). Grade2 and more fatigue was seen in 19% of CMF and 40.9% of FAC group patients (Chi-square P = 0.02). High risk neutropenic sepsis was seen in 2.4% of CMF patients and 6.8% of FAC patients (P = 0.58). One patient (2.3%) died due to neutropenic sepsis in FAC group and none in CMF group. Significant anemia requiring transfusion was seen in 43.1% patients of FAC group and 16.6% of CMF patients (P = 0.007).

**Table 4:** chemotherapy induced toxicities

Toxicity	Frequency (%)
Alopecia	44 (51.2)
Skin/Nail discoloration	44(51.2)
Emesis (Grade2 and more)	18 (20.9)
Mucositis(Grade3 and more)	8(9.3)
Nausea (Grade3 and more)	5(5.8)
High risk febrile neutropenia	3(3.5)

Additionally 12 patients (10.2%) had lymph edema of the upper limb on affected side.

Two patients had radiation pneumonitis, one recovered and another died. Three patients had wound infection.

### Response

Of 118 patients, 33 (28%) relapsed, 52 patients (44.1%) were in remission, 9 (7.6%) had progressive disease. Twenty-four patients (20.3%) had no follow-up.

### Survival

**Figure 1:**
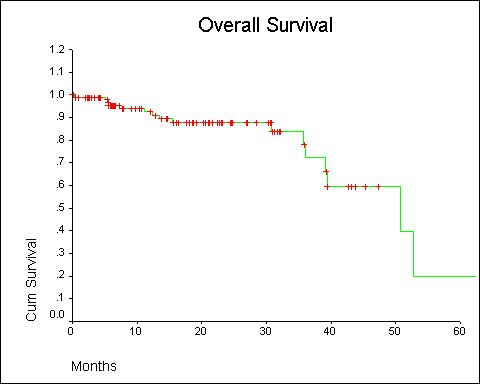
Overall survival.

**Figure 2:**
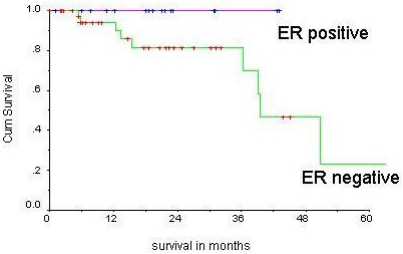
ER status and survival.

Median Survival for the whole cohort was 50.8 months (95% CI 30.7, 70.8). The 3 year survival was 70 percent (Figure 1). ER positive patients did not reach the median survival (Figure 2), whereas for ER negative patients it was 39.4 months (P value = 0.05). Median survival in post menopausal women was 39.4 months and while it was 50.8 months in premenopausal women (P = 0.84). Median survival for CMF chemotherapy group was 50.8 months and FAC group did not reach median survival (P = 0.50).

### Patterns of relapse

Of 33 patients, who had relapsed, 48.4% had skeletal metastases, 27.2% had lung metastases, 12% had liver metastases and 9% had brain metastases. Chest wall recurrence was seen in 33.3% patients and 21.2% patients had lymph nodal relapse.

## Discussion

In this study we found that MRM was the most common primary surgical option for breast cancer. The adjuvant chemotherapy protocols CMF and FAC regimes have shown equivalent survival rates. CMF was less toxic. ER positive patients had superior survival. The compliance of i.v. chemotherapy was good; however the duration of hormonal therapy was suboptimal. Half of the patients did not have data on receptor status and one third on axillary lymph nodal status. Most tumors were receptor negative. The 3 year overall survival was 70%.

Breast conservation surgery (BCS) followed by radiotherapy was shown to be equivalent to MRM in survival [5,6]. Therefore, BCS has become the standard of care for early breast cancer in developed countries [7], although some regional differences in the use of this treatment have been reported [8]. By contrast, MRM was the preferred primary surgical treatment for operable breast cancer in India, including in major urban centers. A study from Delhi showed only 11.3% underwent BCS, while MRM was performed in 88.7% patients [9]. Even in affluent non-western countries this seems to be the case [10]. Access to radiotherapy facility was shown to influence the use of BCS [11]. In the present study, only 6.3% patients underwent breast conservation surgery. Generally, the axillary dissection for most patients was inadequate, as exemplified by the fewer number of nodes examined. Further more, the pathology reports were lacking on pT size and histological grade. These two are very crucial information for optimal treatment of breast cancer. The advanced stage and lack of radiotherapy facility are possible reasons for the infrequent use of BCS.

The EBCTG meta-analysis published in 1992 reported that the adjuvant chemotherapy reduced the annual odds of recurrence by 40% in patients younger than 50 years of age with ER negative tumors and by 33% in those with ER positive disease [12]. In patients above 50 years, adjuvant chemotherapy had reduced the annual odds of recurrence by 30% in ER negative tumors and by 18% in ER positive tumors. Overall, poly chemotherapy significantly reduced the annual odds of recurrence and death by 24% and 15% respectively. Anthracyline based regimens resulted in a survival benefit when compared to CMF-like regimens, with improvement in annual odds of recurrence and death of 11% and 12%, respectively. Toxicity profile is different for CMF and anthracycline based regimens. Various toxicities were more with FAC than CMF in elderly [13]. FAC regimen is costlier than the CMF; the cost becomes even more, if the cost of supportive treatment for toxicities are taken in to consideration. For these reasons, we have preferred CMF for poor, elderly patients. The compliance with either chemo regimen was good. Expectedly the FAC regimen was more toxic and the median survival between the two groups was not statistically significant. To demonstrate 10% difference between the two groups would require a large sample size.

Estrogen receptor is a well established predictive and prognostic factor in breast cancer. The recent international consensus on treatment for early breast cancer [14], the panel affirmed that the first consideration was endocrine responsiveness and suggested categorization of breast cancer in to endocrine responsive, endocrine non responsive and tumors of uncertain endocrine responsiveness. Therefore, testing for estrogen and progesterone receptor status is critical to plan optimal treatment for breast cancer.

Estrogen receptor positive rates were reported to be lower in Indian patients than those in western countries. A study from Mumbai, found ER and PR positive rates of 32.6% and 46.1% respectively [15]. Additionally, not all patients in India undergo the hormone receptor testing, as shown by a study from Delhi; only 35.5% patients had receptor testing [9]. In India, there is a concern about rising incidence of breast cancer in young women; premenopausal women comprised 50% of the cohort in an urban study [9]. However in the present study the premenopausal women formed one-fourth of the cohort. This suggests that the breast cancer in young Indian women may be an urban phenomenon.

In our study, only 48.3% Patients had hormone receptor testing. The mean duration of compliance with tamoxifene was 1.3 years. Even in developed countries the use of Tamoxifene was found to be suboptimal at community level [16]. Nonetheless, the reasons for this poor compliance in our patients require investigation and improvement.

Survival rates in breast cancer patients have been reported from some registries in India. Five-year overall survival rate in the Bangalore population based registry was 42.3% [17]. The Madras Metropolitan Tumor Registry reported survival rates of 80%, 58% and 48% at 1 year, 3 year and 5 years respectively [18]. A study from Kerala showed five year survival rate of 40% [19]. In the present study, the overall survival rate at 3 years was 70%, demonstrating the feasibility of achieving comparable results at community level by improving the infrastructure.

## Conclusion

To move forward in the breast cancer care, breast cancer physicians in India face major challenges. Some of them are lack of information on histological grade of the tumor, pT size, axillary lymph node clearance and receptor status. The skills required by surgeons and pathologists to address these issues can be imparted by conducting workshops and Continuing Medical Education programs. On chemotherapy front, Taxanes and the monoclonal antibodies are unlikely to be used at community level in India, in the foreseeable future. The sequential adjuvant hormonal treatment, with falling prices of aromatase inhibitors, is promising. Therefore, it is necessary for the oncology community in a poor country like India to develop minimum practice guidelines for breast cancer management.

## Competing interests

The author(s) declare that they have no competing interests.

## Authors' contributions

**SK**: Designed the study, supervised the data collection, analysis and writing of the manuscript.

**KRM**: participated in designing the study and critically the paper

**RRPV**: contributed to the study design and write up

**MY**: contributed in writing up.

**AYL**: reviewed the manuscript critically

**PBV**: contributed in writing up

**SKVS**: Data analysis and critically reviewed the paper.

All authors read and approved final manuscript.
